# Machinability of Vitrified Semi-Finished Products: Chip Formation and Heat Development at the Cutting Edge

**DOI:** 10.3390/polym17192681

**Published:** 2025-10-03

**Authors:** Jannick Fuchs, Yehor Kozlovets, Jonathan Alms, Markus Meurer, Christian Hopmann, Thomas Bergs, Mustapha Abouridouane

**Affiliations:** 1 Institute for Plastics Processing in Industry and Craft, RWTH Aachen University, 52074 Aachen, Germany; 2 Manufacturing Technology Institute MTI, RWTH Aachen University, 52074 Aachen, Germanyinfo@mti.rwth-aachen.de (T.B.);

**Keywords:** vitrimers, vitrimeric resin, 4-aminophenyl disulfide, vacuum infusion, carbon fibre reinforced polymers, carbon fibre composites, chip formation, manufacturing technology, orthogonal cutting

## Abstract

Fibre-reinforced composites are facing new challenges in the context particular in sustainability and recyclability. Vitrimers could be useful as new matrices to support the increase in sustainability. Due to their high strength, which is comparable to that of thermosets often used in composites, and their covalent adaptive networks, which make them reshapeable for scaled-up manufacturing and recycling purposes, they are very useful. Orthogonal cutting is used for precise reshaping and functional integration into carbon fibre reinforced plastics. Vitrimers could improve processing results at the cutting edge as well as surface quality thanks to their self-healing properties compared to brittle matrices, as well as enabling the recycling of formed chips and scrap. This study showcases the manufacturing of a carbon fibre-reinforced vitrimer using 4-aminophenyl disulfide as a hardener, with vacuum-assisted resin infusion. The temperature of chip formation and the cutting parameters are then shown for different fibre orientations, cutting widths and speeds. The observed cutting forces are lower (less than 140 N) and more irregular for fibre orientations 45°/135°, increasing with cutting depth, and fluctuating periodically during machining. Despite varying cutting speeds, the forces remain relatively constant in range between 85 N and 175 N for 0°/90° fibre orientation and 50 N and 120 N for 45°/135° fibre orientation, with no significant tool wear observed and lower-damage depth and overhanging fibres observed for 0°/90° fibre orientation. Damage observation of the cutting tool shows promising results, with lower abrasion observed compared to thermoset matrices. Microscopic images of the broached surface also show good quality, which could be improved by self-healing of the matrix at higher temperatures. Temperature measurements of chip formation using a high-speed camera show a high temperature gradient as cutting speeds increase, but the temperature only ever exceeds 180 °C at cutting speeds of 150 m/min, ensuring reprocessability since this is below the degradation temperature. Therefore, orthogonal cutting of vitrimers can impact sustainable composite processing.

## 1. Introduction

Thermosets are used because of their high strength, high temperature and creep resistance, as well as their long term durability [1,2,3]. Epoxy resins as a subgroup of thermosets are extensively applied in aerospace, automotive and electronic applications due to their relatively low price with good processing performance, chemical stability, and the ease to introduce reinforcement materials [4,5,6]. The reinforcement of epoxy resins with fibres (e.g., carbon fibres) can increase the mechanical performance of parts by up to two orders of magnitude, and it is used primarily in light-weight applications [2]. The application of bi- or multi-directional reinforcement with 0°, 90° woven textiles allow a more isotropic reinforcement of the final part such as in chassis in automotive applications, where the loads are not unidirectionally distributed.

Fibre reinforced parts with a thermosetting matrix cannot be mechanically recycled, so that the mechanical properties are retained in a new product made from the recyclate because of the permanent molecular bonds forming during curing [7,8]. Vitrimers were introduced as promising alternative to typical thermoset applications, offering a potential for repair, reuse and recycling (3R) to traditional thermoset applications [9,10,11,12,13]. Vitrimers possess dynamic covalent bonds forming a dynamic covalent network (CAN) when polymerized, allowing a reconfiguration of the polymer network after curing [14,15,16,17]. This property possibly enables the mechanical recycling of thermosetting materials and possible fibre recovery [18,19]. The recent investigation of vitrimers in polymer processing shows processing limitations in specimen sized parts and does not reflect postprocessing steps such as machining [20,21,22,23,24]. Parts with fibre reinforcement are commonly produced via hot-pressing of imine- and ester-based vitrimers as well as fibre reinforced disulfide-cross-linked epoxy vitrimers manufactured by compression resin transfer moulding and vacuum-assisted resin infusion [21,25,26,27,28,29]. Disulfide-crosslinked epoxy vitrimers applied in this study use the curing agent Bis-(4-14 aminophenyl)-disulfide (4-AFD), which contains dynamic molecular bonds (disulfide bonds) but reacts with the epoxy resin by an catalyst free amin reaction identical to the conventional curing mechanisms [30,31,32,33,34]. The compatibility of 4-AFD with conventional epoxy resins as well as the stoichiometry has been studied in previous research, and we replicated it here [30,35]. However, the material processing focuses on vacuum-assisted resin infusion (VARI) as a process with good reproducibility due to processing parameters, where previous studies focused on wet filament winding [30,35].

### 1.1. Machining of Fibre Reinforced Plastics (FRP) and Vitrimers

Often described as the simplest machining condition, the orthogonal cutting operation is frequently used to study and research the basics of material removal processes. Thanks to its relative simplicity, the method is easy to set up and operate, and it does not require a large amount of material for experiments. In addition, the orthogonal cutting method allows isolating different cutting mechanisms and phenomena during the machining process to study them separately. An example of this would be the chip forming, temperature development, and cutting force measurement. In addition, the results obtained during orthogonal cutting can be further extrapolated for other cutting operations, such as turning, drilling and milling, to predict the behaviour of the material [36,37,38]. Considering these advantages, the orthogonal cutting operation was implemented in this work to study the machinability of vitrimere-based FRP.

Postprocessing of thermosets after curing is challenging because of high brittleness, the materials anisotropy and cutting forces can result in high tool wear, and the parts are required to be manufactured in the near-shape form, which complicates or limits the production [39]. The need for machining of thermoset CFRPs stems from increasing demands for dimensional tolerance [40], complex features such as pockets or precise edges [40,41], assembly requirements, [40] or high-precision surface finishing [42]. In comparison, the thermoplastics are less mechanically strong and less thermally or chemically stable. They, however, show a higher degree of impact strength and damage resistance compared to thermosets, as well as excellent formability and weldability, requiring shorter processing time and ability to be recycled more easily. It is also possible to use carbon fibres to reinforce other materials, such as ceramics and concrete, but it is less common [37].

Because of their relative novelty, the machinability of vitrimer plastics as well as composite materials with vitrimer-matrix has not been well researched yet. Vitrimer polymers have not been widely implemented in the industry as of date. Despite this however, vitrimer plastics have a lot of potential in wide range of industry branches because of their exceptional combination of mechanical properties similar to thermosets, as well as recyclability and malleability at higher temperatures similar to thermoplastic polymers, for example in aerospace and automotive industry, sports equipment and construction [6,43,44].

The machinability of vitrimer plastics is highly dependent on the temperature of the material. In the work of [44], in which the machining of vitrimer plastics was compared with machining of pentaerythritriol-based epoxy (PER) and a polycarbonate (PC) material, by comparing the cutting force during the orthogonal cut operation as well as the resulting surface quality of machined samples. It has been shown that the vitrimer performs better in comparison to the PER at room temperatures. The main force component F_c_ during cutting decreases with the increase of temperature for all materials. However, the PC and vitrimer plastics show high deviations of cutting force at higher temperatures. It has been shown that increasing temperatures not only initiate the degradation of vitrimer plastics, but also hinder their unique self-healing or consolidation properties [44]. The vitrimer hardener 4-AFD used in this study exhibited a decomposition temperature of 250 °C with comparable matrix systems, as evidenced by prior research employing thermogravimetric analysis. A degradation onset can occur as early as 210 °C with matrix systems with a lower molecular weight [35]. Degradation of the CAN during manufacturing or a subsequent process can also be visualized by the degradation of surface quality, which is reported in [45].

### 1.2. Chip Formation and Recyclability

The cutting of FRP materials differs significantly from metals because of their anisotropic microstructure. Material separation in thermoset FRPs occurs mainly through breaking and cracking mechanisms. Tool loads cause fibre failure due to pressure, bending, and shearing [38,46]. With fibres perpendicular to the tool, separation occurs by bending and inter-fibre shearing, producing cracks in matrix material. At higher angles, fibres tend to glide over one another, which is more typical for thermoset matrices [38,47].

The shape of the chips forming during the machining of FRP is mostly discontinuous and fragmented, with their size decreasing with increased orientation angle as the angle increases the discontinuous dust chips start to form instead of the fragmented chips [48].

Mechanical loads are not the only factors affecting the machining of the FRP. Due to the thermal properties of the FRP matrix materials, the temperature development must also be closely monitored. At temperatures close to the glass transition, the matrix in the composite will start to degrade, which will lead to further defects, such as delamination during the machining, and reduce strength of the FRP part. Additionally cutting temperature will also influence the interaction between the FRP and tool surface, which will also affect the wear of the tool [36,49,50,51,52]. Initially, most heat flows into the tool. Later, as the tool heats up, more heat enters the workpiece causing temperature build-up [38]. Because of low heat conductivity of the matrix, heat rise is mainly surface-localized. Even at high speeds (300 m/min), when surface temperatures exceeded T_g_ the subsurface temperatures stayed low (104 °C) [49].

Not all heat produced during the machining flows in the tool or the workpiece. A significant part is removed together with the chip. Heat removal depends on the operation: during milling/turning approximately 45–95% of heat tends to be removed, while during drilling only 25–37%—chip transport through drill flutes causes secondary heat transfer back to the tool [37].

The mechanical recycling of chips and the reduction of waste from the FRP is motivated by the low-level thermal or chemical alteration of the matrix material [53,54]. The process of pyrolysis has been shown to cause the degradation of the functional chemical bonds within the matrix or sizing, thereby rendering them unusable [55]. Solvolysis processes have been designed to detach the matrix from the fibres, thereby hindering the full utilisation of the advantages of vitrimeric resin [56,57]. Reconsolidation can only be achieved through re-impregnating the fibre afterwards. The process of mechanical comminution results in the production of fibres that contain matrix residues. These residues can be re-integrated into a composite material through the reinstatement of CAN [58,59].

However, the mechanical recycling or orthogonal cutting of the CFRP also results in a significant reduction in fibre length [53]. This reduction, concomitant with a decline in mechanical properties, often results in the utilisation of fibre waste currently being used as filler. For instance, they are used as fillers in concrete mixtures, either to substitute for sand or to enhance its strength and rigidity [60,61]. The utilisation of vitrimers, which possess the capacity for matrix reuse due to the CAN, has the potential to address a significant gap in the recycling of short-fibre-reinforced materials. To achieve this objective, it is necessary to investigate the manufacturing and processing parameters, including cutting force and temperature of chip formation, and their influence on vitrimer semi-finished products and chips. This is addressed in the presented study and will lead to matrix-friendly processing of vitirmers and facilitate the reuse of the CFRP materials.

The vitrimeric FRP materials show high potential for application in industry. However, there is still insufficient information about their behaviour during machining operations. In this study, a total of 30 vitrimeric CFRP samples with different fibre orientation were prepared and machined using the operation of orthogonal cutting, using varying cutting parameters. The machinability of vitrimeric CFRP parts was characterized by measuring the force components and temperature in the cutting zone during the machining operation, as well as capturing the chip formation during cutting with high-speed camera and later analysing the quality of machined surface on the samples. This allows obtaining the basic understanding of the behaviour of vitrimeric CFRP during cutting and how it could affect the machining process during manufacturing. This understanding outlines the possible avenues for further research and sustainable production of vitrimeric CFRP parts.

## 2. Vitrimer Specimen Preparation

### 2.1. Vitrimeric Materials

The presented study uses the epoxy resin EPIKOTE Resin 04976 provided by Westlake Epoxy GmbH, Duisburg, Germany with the vitrimeric curing agent 4-AFD. Previous studies demonstrated the vitrimeric behaviour of the cured material using 4-AFD as curing agent [30,35]. Additionally, multiple material properties such as gelation times, temperature dependent processing viscosity, and resulting mechanical and thermo-mechanical properties are reported in previous works. Therefore, the measurements were not repeated here [30]. Because of the high melting temperature (84 °C) of the 4-AFD curing agent, the curing agent must be molten before mixing with the epoxy resin. Therefore, the curing agent and epoxy resin were preheated to 90 °C in a vacuum oven and mixed manually for 2 min. A weight ratio resin to curing agent of 69.93:30.07% was chosen according to the literature [30,35].

### 2.2. Infusion Process of Carbon Fibre Reinforced Fabrics

**Figure 1:**
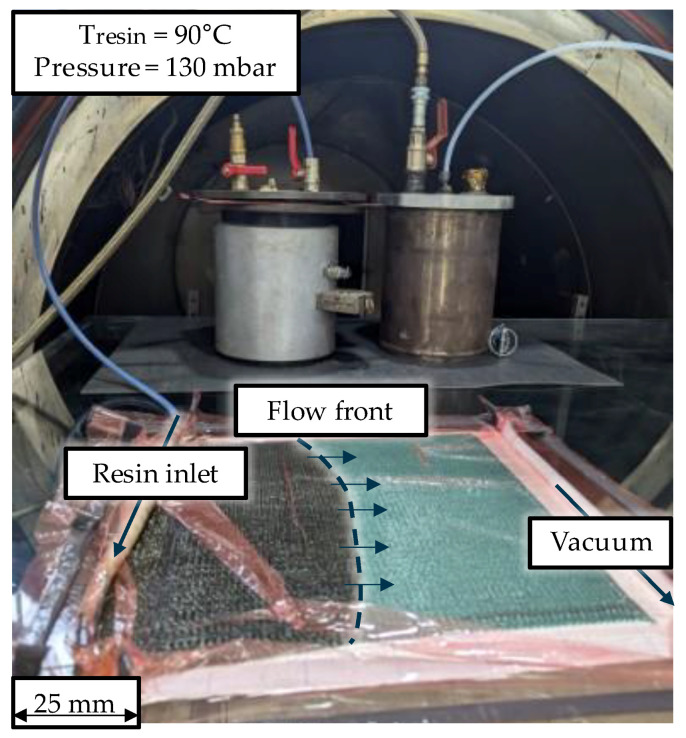
Vacuum assisted infusion of vitrimeric resin in an autoclave.

The fibre reinforcement used in the study is a carbon fibre woven with [0/90] biaxial fibre orientation, Sigratex CW245-TW2/2-SQ120, SGL Carbon SE, Wiesbaden, Germany. To obtain a laminate thickness of roughly 2 mm, six layers with a dimension of 300 × 300 mm^2^ were draped onto a glass plate, coated with release agent Frekote 700 NC, Henkel AG & Co. KG, Düsseldorf, Germany. The plate was sealed with vacuum bagging and pre-heated to 70 °C in an autoclave, Scholz Maschinenbau GmbH & Co. KG, Coesfeld, Germany, to ensure a heated surface of the glass for a better resin flow. The vitrimer resin was infused with a vacuum of 130 mbar (Figure 1). Since the viscosity of the resin is below 60 mPas above 80 °C, but over 100 mPas at 30 °C [29], the infusion process of Plate 2 is additionally assisted by a hot air blower across the resin inlet and in direction of the flow front on both sides of the plate, with temperature setting at 120 °C. Plate 2 exhibited a filling time of 25 min resulting in an average volume flow rate of approx. 0.13 mL/s. After the infusion, the specimen were cured in the autoclave for one hour at 90 °C and afterwards for two hours at 140 °C under constant vacuum, but without external pressure. All temperature ramps were set to 2 K/min. Two plates were manufactured with this method. Minor defects were observed after the infusion of the first plate, which later on was confirmed by analysis of resulting porosity and fibre volume content.

### 2.3. Quality Analysis of Fibre Reinforced Vitrimeric Specimens

**Figure 2:**
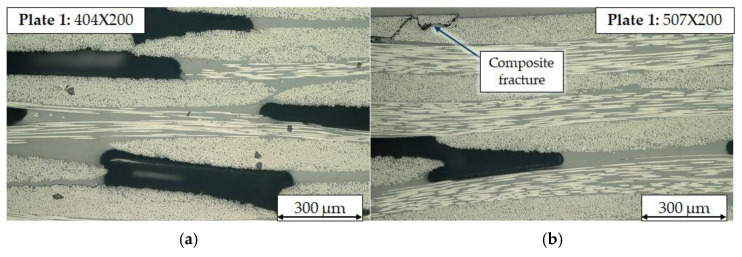
Cross section with 200× magnification of Plate 1 with various defects: (**a**) high porosity and resin rich areas, (**b**) porosity and composite fracture at edge of the specimen.

The impregnation quality of the woven material is defined by the achieved fibre volume content (fvc) as well as the pore content and pore sizes. To acquire information about impregnation quality five random selected cross-sections from the edges of the manufactured plates were extracted measuring 10 × 2 mm^2^ and were examined at 200× magnification using VHX-600 light microscope, Keyence, Oska, Japan. Figure 2 shows exemplary various defects that were visible after manufacturing of Plate 1. Pores with big sizes between woven rovings indicate that the middle layers of the composite were not completely infused by the vitrimer matrix (Figure 2a). High pore content, especially pores of high volume can cause varying cutting forces and uneven chip formation. Also fractures of the composite occur on the edges of the composite, which can be caused by extraction of the samples, indicating a higher potential for delamination. The occurrence of delamination will decrease part quality after mechanical post processing of CFRP but can be avoided by adapted cutting speeds.

**Figure 3:**
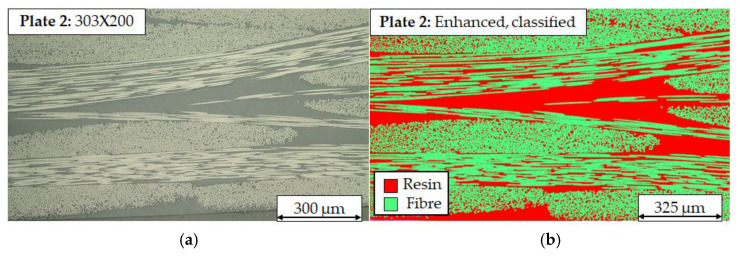
(**a**) Cross section with 200× magnification of infused Plate 2 with low porosity (**b**) classification of the same cross section using WekaSegmentation trained model.

In comparison to Plate 1, exemplary five cross section of Plate 2 show a higher impregnation quality, especially in overall visibility of pore content. The density of rovings from the woven material appears similar, even though Plate 2 shows more resin rich areas. This can be explained by overall lower viscosity of the resin during the infusion process due to the hot air assist, resulting in overall better resin flow (Figure 3a). It can be concluded that additional process heat can stabilize impregnation quality with vitrimeric matrices with higher viscosity.

A quantification of resulting fvc and pore content was conducted with five cross-sections of each manufactured plate using the open-source image software ImageJ (Fiji V2.9.0) with the WekaSegmentation (V4.0.0) plugin. The classification of images is based on a training dataset, which was produced with a reference CFRP with a thermoset matrix produced by VARI. The images were classified into three categories: resin, fibre, and pores. This was achieved within a threshold, determined by their greyscale value, and subsequently calculated for the respective area fraction of the class. As illustrated in Figure 3b, the classification and precision, thereof, were demonstrated for a representative cross section of Plate 2.

**Figure 4:**
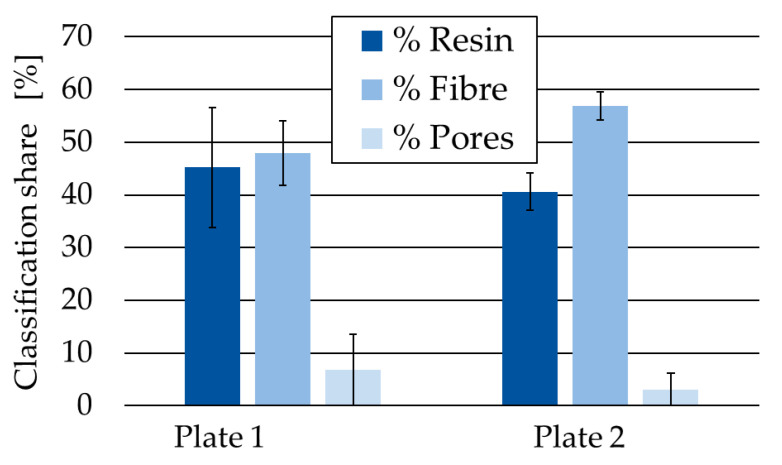
With WekaSegmentation classified percentage of resin, fibre and porosities of infused carbon fibre plates.

The analysed fvc and pore content within each plate confirm the qualitative assessment of the microscopic images, showing a low impregnation quality of the samples in Plate 1 (Figure 4). Plate 1 exhibits a fvc of 47.95 ± 6.1% with a pore content with a minimum of 0.4% and a maximum of 24.6% in between the samples. Since the pore content and resin content in Plate 1 shows high fluctuation in between each cross section, the variance in the resulting laminate properties could influence its machinability. Plate 2 results in a more homogenous fvc of 56.9 ± 2.7% and a pore content of 3.1 ± 3.0% (Figure 4). High standard deviation of porosity stems from a few pores with bigger pore size. This irregularity may be attributable to the extraction of samples from the edge of the infused plates. Since infusion of Plate 2 with vitrimeric resin results in better laminate properties, specimen of Plate 2 were used in machining operations in this paper.

### 2.4. Specimen Preparation for Orthogonal Cutting

After infusion with vitrimeric resin, the plates were cut into the specimens with dimensions 20 × 40 mm^2^ using the precision saw Discotom-100, by Struers GmbH, Willich, Germany. In total, 30 specimens were prepared for the experiment, with two types of reinforcing fibre weave orientation in the material—perpendicular [0|90°] to the direction of the cut and diagonal to the direction of the cut [+45°|−45°]. Before the experiment, one side of the specimens was coated with a layer of a black heat-resistant lacquer by Peter Kwasny GmbH, Gundelsheim, Germany, in order to enhance the thermos-optic value for thermal imaging during the cutting process.

## 3. Orthogonal Cutting of Fibre Reinforced Vitrimeric Specimens

### 3.1. Orthogonal Cutting of Fibre Reinforced Vitrimeric Specimen

**Figure 5:**
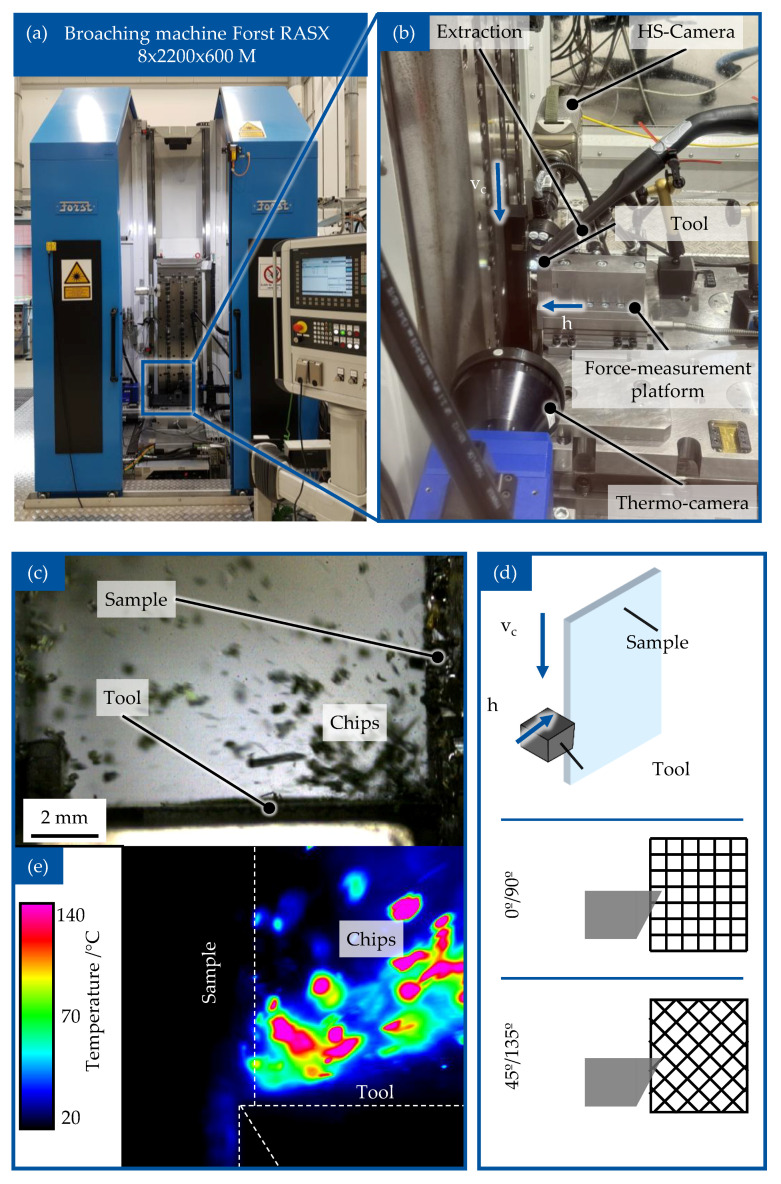
Experiment setup for orthogonal cutting of Vitrimer-CFRP: broaching machine (**a**) and cutting setup with themo camera (**b**), chip formation recorded with high-speed camera (**c**), schematic view of orthogonal cutting of the sample (**d**) and temperature development during chip formation (**e**).

To examine the machinability of Vitrimer-CFRP, an orthogonal cutting operation was used. The experiment was performed on the broaching machine Forst RASX 8 × 2200 × 600 M/CNC. Because of the expected high tool wear during the machining of CFRP materials, the polycrystalline diamond (PCD)-cutting tools SCGW 09T3AT00725A1VE006 DA1000 manufactured by Sumitomo Electric Hartmell GmbH, Willich, Germany, were chosen for this work and are depicted in Figure 5a.

The cutting setup and positioning of the high-speed camera and the thermo camera are shown in Figure 5b. During the experiment, the specimens were installed and secured into the holder able to move in the vertical direction with varying speeds from 10 m/min up to 150 m/min. The cutting tools were installed into the custom-made holder to enable moving along the horizontal plane. By changing the position of the tool, an undeformed chip thickness (h) can be set. To analyze the machinability of vitrimer-CFRP, the orthogonal cutting operation was performed with varying cutting parameters, such as orientation of the reinforcing fibres, cut width, and cut speeds. The orientation of the reinforcing fibre depending on motion of tool and test specimen is shown schematically in Figure 5d. The cutting parameters were chosen according to literature [38] to observe the behaviour of vitrimeric-FRP samples under industrial manufacturing conditions (Table 1).

During the cutting process, the measurements of cutting force components—cutting force F_c_ and normal cutting force F_cN_ were captured using the force measurement plate KISTLER Type 9257B, to which the holder with the cutting tool is attached (Figure 5b). During the cutting operation, the change of the temperature in the working zone was captured using thermal camera Flir SC 7500 (FLIR Systems GmbH, Frankfurt, Germany). The process of material separation was captured in detail using a high speed camera Phantom v7.3 (Vision Research Inc. Wayne, NJ, USA). Pictures form chip formation and their coloration due to thermomechanical load can be observed in Figure 5c. Due to the danger of emission of harmful substances, such as chemical smoke and dust particles, the chips and other separated material were captured during the experiment using an industrial vacuum cleaner, BOSCH GAS 35 H AFC (Robert Bosch Power Tools GmbH, Leinfelden-Echterdingen, Germany), in accordance to the safety guidance.

### 3.2. Cutting Force Measurements

**Figure 6:**
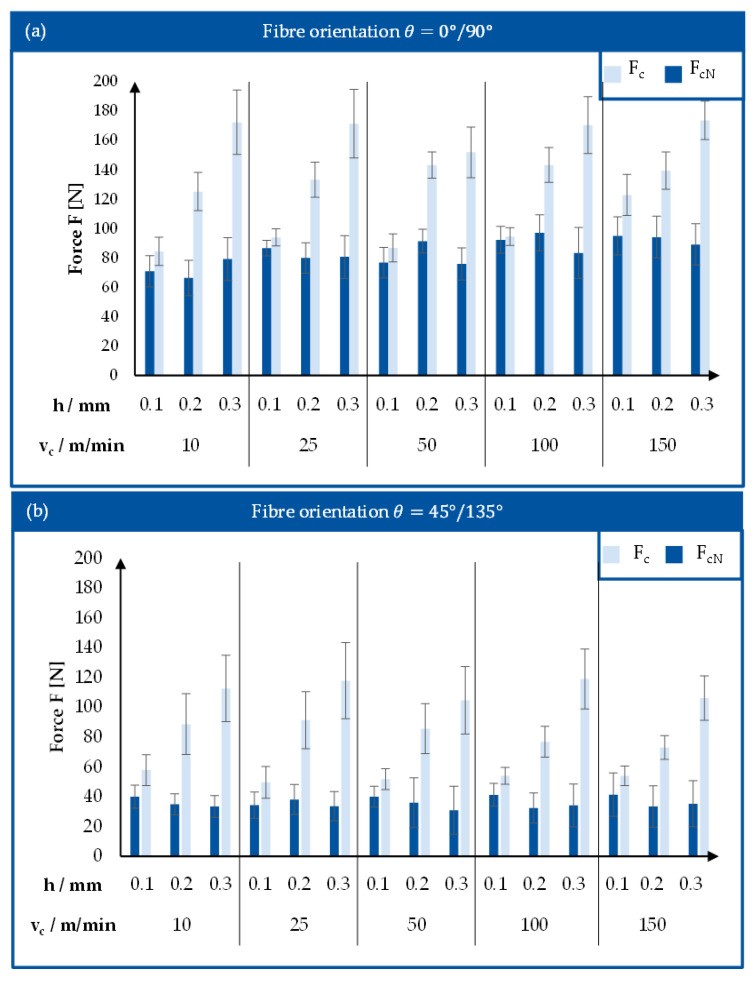
(**a**) Cutting force components during the orthogonal cutting of Vitrimer-CFRP with fibre orientation *θ* = 0°/90°. (**b**) Cutting force components during the orthogonal cutting of Vitrimer-CFRP with fibre orientation *θ* = 45°/135°.

Figure 6a,b presents the average values of F_c_ and F_cN_ during the orthogonal cutting of Vitrimer-CFRP. Comparing the graphs shows that the values of cutting forces are noticeably lower when cutting the samples with the diagonal orientation of reinforcement weave. This may be caused by the difference in chip building mechanism from bending of the fibres to shearing and pressing. The cutting force F_c_ is always higher compared to the normal cutting force F_cN_, independent of the orientation of fibre in the material or process parameters. The cutting force F_c_ in-creases with higher values of cutting depth h for both types of fibre orientation. In comparison, the values of normal cutting force F_cN_ remain stable with relatively low deviations. There is, however, a small decline of normal cutting force with an increase of cutting depth is observed.

**Figure 7:**
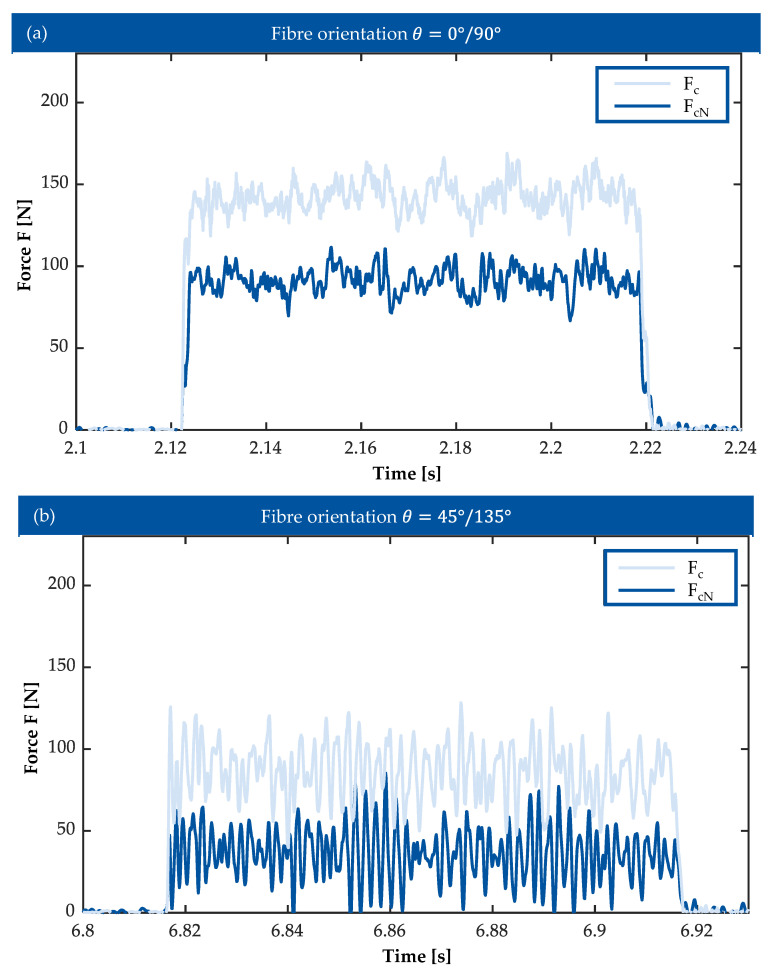
(**a**) Cutting force during the cutting process: sample with fibre orientation θ = 0°/90° (v_c_ = 50 m/min, h = 0.2 mm). (**b**) Cutting force during the cutting process: sample with fibre orientation *θ* = 45°/135° (v_c_ = 50 m/min, h = 0.2 mm).

The values of cutting force do not remain constant during the cutting process, as is shown on Figure 7b. Apart from the small noise of the measurements, a noticeable periodic wave is present in the values of both normal and cutting forces. In addition, the diagrams show higher level of noise for the samples with diagonal orientation of fibres. This phenomenon is not unusual for machining of CFRP material—the signal wave indicates the interchanging interaction of the cutting tool with matrix material and fibres.

### 3.3. Temperature Measurements

**Figure 8:**
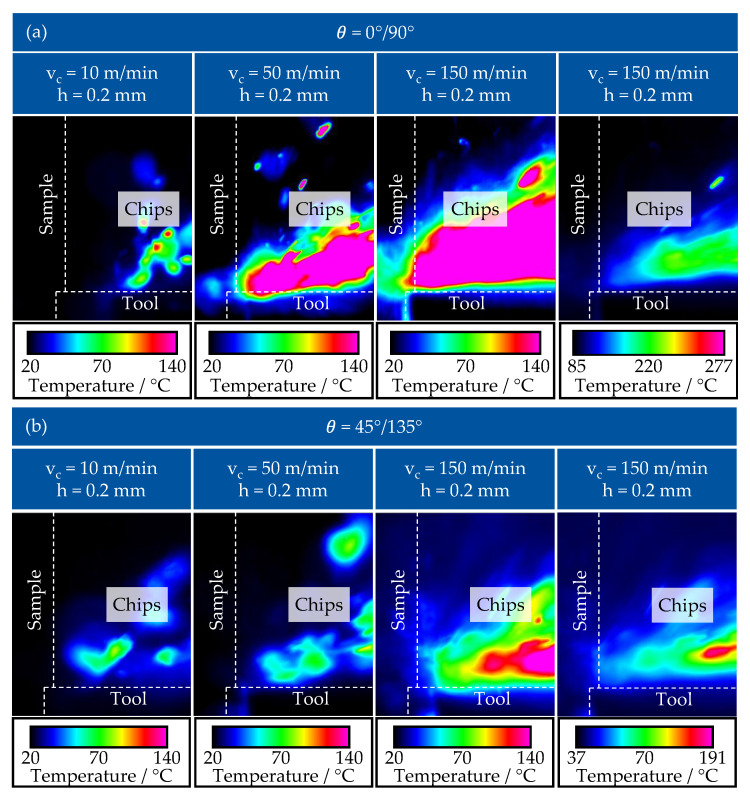
Temperature of the chips during the cutting process with a fibre orientation of (**a**) *θ* = 0°/90° and (**b**) *θ* = 45°/135.

It is noticeable that the temperature in the cutting area is increasing with higher cutting speed for both types of fibre orientation (Figure 8a) 0°/90° and (Figure 8b) 45°/135°. According to the literature [62], a significant part of the heat produced during cutting (approx. 45–95%) is removed from the cutting zone with the separated material, only a slight increase in the temperature of the machined surface is observed—the temperature of the specimen has not increased above the value of 100 °C for both examined fibre orientations. The temperature of the removed material is increasing with higher values of cutting speed—as such, at the cutting speed of 10 m/min the chips do not reach the temperature above 140 °C, while at the cutting speed of 50 m/min the temperature increases to the values between 140–180 °C. The similar trend of the temperature increases with increase of width of uncut chip *h* and cutting speed *v_c_* was also observed in different studies of machining of CFRP [52,63,64]. An et al. observed temperatures of 160 °C at cutting speeds of 150 m/min for a fibre orientation of 90°, consistent with Figure 8a right [64]. Changing the fibre orientation lowers the temperature of chip formation, e.g., for 45° (140 °C) and 135° (85 °C), consistent with Figure 8b right. The processing of the vitrimer thus offers no decrease in temperature development during chip formation, as this is a fibre-dominated property [64].

For higher cutting speeds and depths as well as other machining operations, significantly higher temperatures are recorded during chip formation for thermoset CFRP [46,47,48,64]. The recorded temperature range in the presented study lies under degradation temperature, which was determined in previous studies [30,35]. This implies limited thermal degradation on the surface of the machined vitrimer, preserving the self-healing and reusable properties of the CAN. Chips with said process parameters observed could possibly be used as mass for post-industrial recycling. With further increase of the cutting speed up to 150 m/min, the chips reach the degradation temperature of vitrimer material of more than 235 °C. Degradation onset for the vitrimeric resin was previously determined with a thermogravimetric analysis at 210 °C, with a decomposition temperature at 250 °C, higher cutting speeds would disallow the reuse of chips. Comparable epoxy resin, with anhydride curing mechanism, exhibited a degradation temperature of 300 °C, which would allow higher cutting speeds before degradation occurs [35]. Depending on the rake angle and cutting depth, cutting speeds of up to 300 m/min are achieved in orthogonal cutting of thermoset cfrp [47,64]. The lower degradation temperature of the vitrimer thus limits the possible processing speed.

Because of the separated material in the form of small chips, particles, and dust obscuring the working area and affecting the captured light emission, it is complicated to accurately measure the temperature in the cutting zone using thermal camera. This problem of reducing the effect of dust on the temperature measurement needs to be addressed in the future research.

### 3.4. Chip Formation During Machining

**Figure 9:**
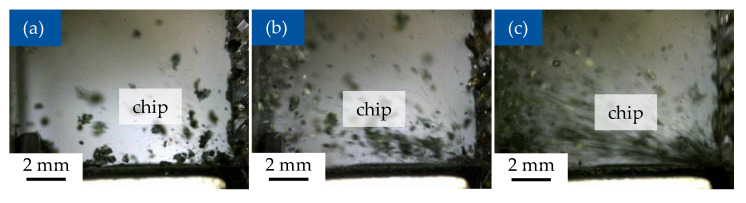
Cutting process, orthogonal fibre orientation 0°/90°, cutting depth h = 0.2 mm; (**a**) cutting speed v_c_ = 10 m/min; (**b**) cutting speed v_c_ = 50 m/min; (**c**) cutting speed v_c_ = 150 m/min.

**Figure 10:**
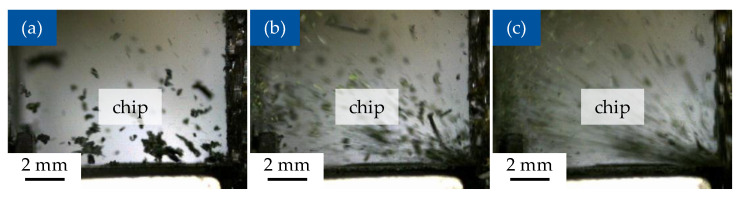
Cutting process, diagonal fibre orientation 45°/135°, cutting depth h = 0.2 mm; (**a**) cutting speed v_c_ = 10 m/min; (**b**) cutting speed v_c_ = 50 m/min; (**c**) cutting speed v_c_ = 150 m/min.

At the of cutting speed of 10 m/min, the removed material is separated in the form of relatively coarse particles, which consist of the carbon fibres with the vitrimer matrix. The form of particles changes with increase of the cutting speed, with the size of the chips reduced until reaching fine dust at the cutting speed of 150 m/min (Figure 9 and Figure 10). This results are according to the machining for thermoset CFRP [48].

The recordings of the machining process also show a change of colour from black to dark green of removed material e.g., in Figure 10b, which is characteristic for the debonding of disulfide bonds of the CAN or alternatively a phase transformation in the material due to the temperature increase. However, during the experiments performed there were no opportunities present to gather the removed chips safely for analysis, so the material change could not be proven. Additionally, in the testing the colour setting of the high-speed camera was not calibrated, which could also be the cause of the observed extent of the colour change.

### 3.5. Tool Wear

**Figure 11:**
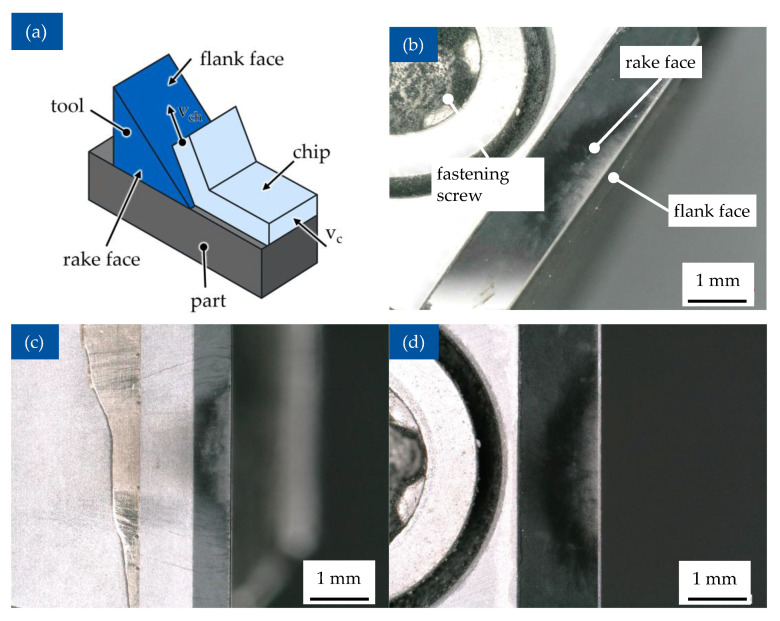
Tool wear: (**a**) schematics of orthogonal cut [67], (**b**) panoramic view, (**c**) flank face, and (**d**) rake face.

During the experiments, one cutting instrument was used for total cutting length of approx. 5 m. According to the literature, machining of CFRP-materials commonly causes intense tool wear—a hard reinforcing fibre often has an abrasive effect on the surface of the tool and can cause a significant change in the microgeometry. A common effect is abrasive wear of rake and flank faces together with rounding of the tool edge [65,66]. Contrary to the expectations, however, there is little noticeable signs of tool wear—only a slight discoloration of the flank and rake face is present, without the expected change of tool geometry (Figure 11). Because of this difference in behaviour compared to thermoset CFRP, this lower abrasion must be attributed to the vitrimer matrix. However, as the fibre causes greater abrasion due to its comparable higher stiffness, the mechanism should be investigated further in future studies.

### 3.6. Microscopic Analysis of Machined Surface

There are several characteristics available to quantify the damage of the edges of CFRP parts after the machining operation. The two main characteristics are the deep damage and the overhang of fibres [38].

**Figure 12:**
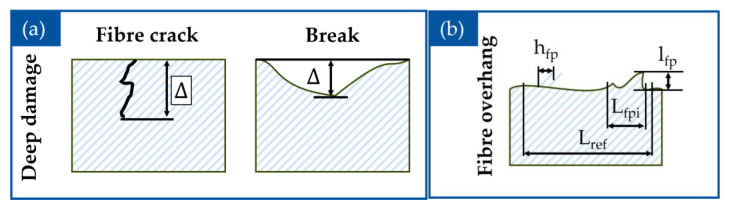
Characterization of surface damage [38]: (**a**) deep damage and (**b**) fibre overhang.

The deep damage of the part is characterized by the maximal depth of the crack perpendicular to the edge of the part, or, alternatively, the maximal surface of the damaged area. The overhang of fibres is further distinguished into the length of the fibres in the main plane of the sample and their height perpendicular to the main plane of the sample (Figure 12).

**Figure 13:**
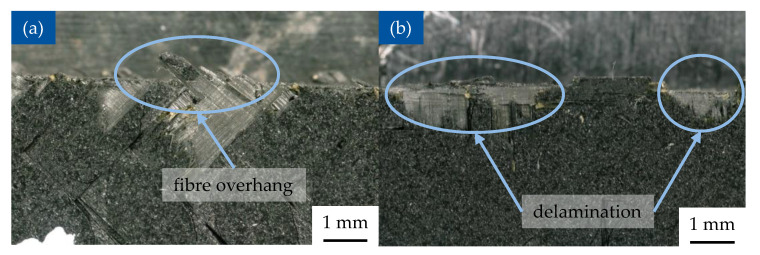
Machined samples exhibiting surface damage: (**a**) fibre overhang with fibre orientation *θ* = 45°/135 and (**b**) delamination with fibre orientation *θ* = 0°/90°.

After machining operations, both sides and machined surfaces of every sample are observed under the optic microscope VHX 5000 by Keyence, Osaka, Japan. Using the software VHX-5000_900F (Version 1.5.1.0) for analysis of digital images the values of fibre overhang as well as the damage depth are measured (Figure 13a,b).

The defects of the machined CFRP parts were classified according to the system of Colligan and Ramulu [38]. After observing the machined samples, the delamination and fiber breakage (Type 1, Figure 13b) as well as the fiber overhang (Type 2 and 3, Figure 13a) were observable.

The delamination defect is caused by the bending of the fibres. The mechanical load acting on the fibre strands may overcome the structural strength of the matrix material bonding the fibres together, which in turn initiates the separation of material layers or singular fibre strands one from another. As the bending increases further, the strands can break after reaching their breaking point (Figure 13b). In his work, Wang [68] determined three different fibre angle dependent cutting mechanisms, which occur during the machining of CFRP. At θ = 0°, the chip formation occurs due to the fracture along the fibre-matrix interface because of bending of the fibres combined with the fracture perpendicular to the fibre orientation. With the increase of θ up to 75°, the cutting mechanism changes to the compression induced shear perpendicular to the fiber axis. The chip is separated from the workpiece through the fracture along fibre matrix interface. At the θ 90° and more, the chip formation and material removal occur because of both in plane and out of plane shear fractures along the fiber-matrix interface, followed by significant fibre deformation induced by the compressive tool load. These results were further confirmed and researched in the study of Abena et. al. [69], where the impact of cutting parameters on the chip formation, cutting force, and damage were determined. The samples with both orientations 0°/90° and 45°/135° show the defects of this type. For the samples with fibre orientation 0°/90°, the primary loads acting on the fibres were pressure and shearing acting perpendicular to the fibres with angle of 90° and along the fibres oriented at the angle of 0, which causes the fibres to bend and break. In the samples with fibre orientation 45°/135°, a part of the fibres was oriented with a smaller angle (45°) to the cutting path, which in turn caused the fibres to break from the part in the form of packets due to the pressure load acting along and bending load acting perpendicular to the fibres. The fibres oriented with a larger angle (135°) were in turn separated from the material by breaking due to the pressure loads applied perpendicular to them by the edge of the cutting tool [38].

If the fibre deformation is insufficient to reach their breaking point, the fibres can then return to their initial position without separating from the machined surface, resulting in fibre overhang (Figure 13a). The defect of this type was more prominent on the samples with fibre orientation 45°/135°. The fibres oriented with the smaller angle (45°) require less deformation to move away from the cutting edge and therefore, often do not reach their breaking point. The samples with fibre orientation 0°/90° also showed defects of this type, but with lesser intensity. While the fibres with orientation of 0° required the leas work to be separated from the material, the crack propagated along the fibres without going deep into the matrix. In addition, the fibres that oriented with 90° and were affected by the pressure, and the shear load perpendicular to them were also bended, but at a shorter length [38].

**Figure 14:**
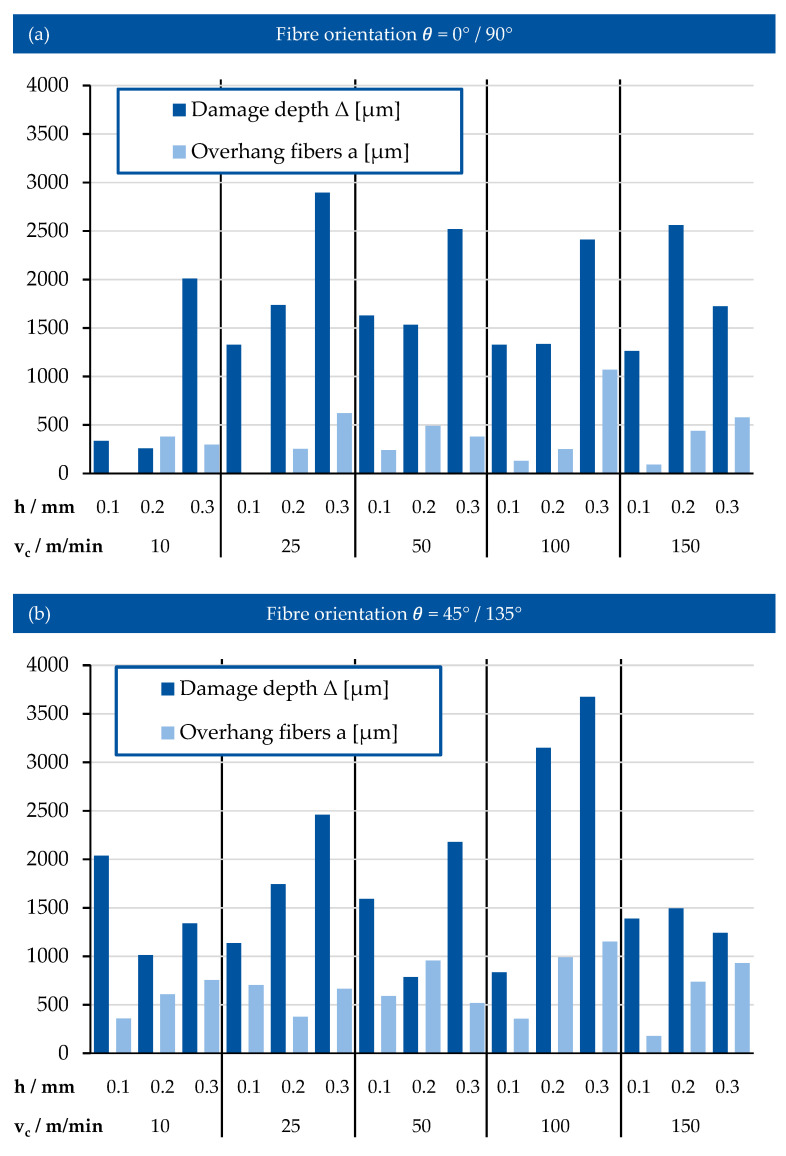
(**a**) Damage depth and overhang length of machined samples with fibre orientation 0°/90°. (**b**) Damage depth and overhang length of machined samples with fibre orientation 45°/135°.

The results of measurement in damage depth and overhang fibres for both fibre orientations and the cutting parameters v_c_ and h are presented in Figure 14a,b.

From Figure 14a for fibre orientation 0°/90°, it is evident that the damage depth increases with increasing depth of cut. The growth lies in the range of 1–1.5 mm. In addition, the damage depth also increases with increasing cutting speed. It should be noted that the increase was observed only for speeds up to 25 m/min. At the cutting speed of 50 m/min and higher, the damaged depth remains relatively constant. A possible cause of increased damage could be the increase of contact area between the workpiece and cutting tool, which leads to higher mechanical loads acting on the material, as is evident from the cutting forces. The high load in machined workpiece may exceed the mechanical strength of matrix and cause crack propagation in the material.

The length of the overhang is observed to be always lower than the damage depth. At the cutting depth of 0.1 mm with cutting speed of 10 m/min and 25 m/min, no fibre overhang was observed. In general, the length of the overhang fibres remains the same with slight increase between approximately 0.1 to 0.5 mm with increase of the cutting depth. This shows that the length of the uncut fibres does not depend on the cutting speed. The defining parameter here is the depth of the cut, which affects how strong the fibres of the composite are bending. The only exception is the experiment with cutting depth 0.3 mm and cutting speed 100 m/min, where the length of the fibres has reached 1 mm.

In comparison, Figure 14b illustrates that the fibre orientation 45°/135° shows distinctly higher values of both damage depth and fibre overhang. Similar to the orientation 0°/90°, the values of damage depth show continuous increase with higher cutting speed. However, at the diagonal orientation, the growth continues up to the value of speed 100 m/min. In addition, at the cutting speed of 150 m/min a step decrease in damage depth is observed, which remains consistent at every depth of cut. This indicates the change in the chip forming mechanism during the cutting operation. Due to the diagonal orientation of the fibres, a part of the shearing and bending load is acting perpendicular to the fibres, which leads to stronger bending of separate fibre strands. This also affects the length of uncut fibres on the machined edge of the sample. The overhang length at fibre orientation 45°/135° has minimal changes with varying cutting speed. There can still be observed the increase of overhang with increasing depth of cut—the length of fibre increases from the values approximately 0.2 mm up to 1 mm.

In comparison to conventional anhydride curing thermosets, the fracture behaviour of vitrimeric materials after initial curing is similar, and it is observed in the different interlaminar fracture modes. The observed similarity leads to the conclusion that the debonding rate of the dynamic covalent bonds is low in comparison to the shear rate in the material. In the literature, the vitrimer parts, reconsolidated from fractured vitrimer, show a higher influence of dynamic covalent behaviour on the fracture mode [45,70,71]. However, it is to determine the degree and mechanism of failure directly contributed to debonding of dynamic covalent bonds. The potential for surface damage to be reduced after machining with vitrimer matrix needs to be addressed in future research. If damage depths exceed the residue matrix on the surface of the specimen, the mechanical properties and surface quality will decrease. Therefore, a careful machining with medium cutting speeds and depths is preferred.

## 4. Conclusions and Outlook

In this study, the behaviour of CFRP with vitrimer, as a matrix material, during machining operation was observed and analyzed with a focus on machinability of the material.

To investigate the machinability of vitrimer CFRP, an experiment of orthogonal cutting with PCD-tool was performed using varying cutting parameters. For the experiment, a total of 30 CFRP material samples with dimensions of 20 × 40 × 2 mm^3^ were used with two different fibre orientations (orthogonal 0°/90° and diagonal 45°/135°). During the experiment, the depth of cut and cutting speed were varied from 0.1 mm to 0.3 mm and from 10 m/min to 150 m/min, respectively. The material separation process and temperature in the work area were captured using high-speed and thermographic cameras. In addition, two components of cutting forces—cutting force F_c_ and normal cutting force F_cN_—were also measured. After the machining operations, each sample was analyzed under the optic microscope to characterize the quality of machined surface. From the analyses, the following key conclusions were derived:

- Cutting forces are lower with diagonal fibre orientation, increasing with cutting depth, but remaining largely independent of cutting speed. Periodic fluctuations occur with stronger signal noise for diagonal fibres. The change of force indicates the different chip forming mechanisms.
- At higher cutting speeds, the chip temperature exceeds 235 °C, while the sample surface remains below 80 °C. Most heat is removed with the chips. Measurements may be affected by the emission from dust particles and need to be considered during the later studies.
- Chip formation depends strongly on cutting speed: large fibre–matrix chips occur at low speeds, while finer particles and dust appear at higher speeds. Colour changes of the removed material may indicate debonding of disulfide cross-links.
- No significant tool wear was observed. Only slight discolouration without geometric changes occurred.
- Surface quality is more affected with diagonal fibre orientation. Damage depth increases with cutting depth due to the increased mechanical loads. For 0°/90° orientation, the damage becomes stable above 50 m/min, whereas with diagonal orientation it increases up to 100 m/min and then decreases sharply at 150 m/min. Fibre overhang is consistently lower than damage depth and mainly increases with cutting depth which affects the bending of the fibres.
- To minimize the surface damage of machined vitrimeric CFRP parts and preserve the recyclability of waste materials, the cutting speed must be set in range of 25–50 m/min with the cutting depth not greater than 0.2 mm

To achieve the sustainability goals for CFRP, the post-processing with machining operations of vitrimers should be further investigated. The presented study shows that the chip formation, based on its discolouration and colour change to green due to the breaking of disulfide bonds, can enable inline characterization of the cutting operation and material degradation. The temperature development and its influence on the recyclability of the chips need to be investigated with thermal cameras in future studies to enable the reuse of production waste. It is conceivable to develop a digital interface that detects the cutting condition by colouring of the chip formation and automatically adjusting cutting parameters to preserve the dynamic bonds in virtimer materials. Ultimately, the self-healing properties can be used for an optimized surface structure and damage repair after machining operations in order to meet mechanical and quality requirements after post-processing.

## Figures and Tables

**Table 1:** Parameter variation for preliminary tests of cutting the Vitrimer-CFRP.

Process Parameter	Unit	Variation
Orientation of fibres to the direction of the cut *θ*	°	0°/90°/45°/135°
Undeformed chip thickness *h*	mm	0.1/0.2/0.3
Cutting speed *v_c_*	m/min	10, 25, 50, 100, 150

## Data Availability

The original contributions presented in this study were included in the article. Further inquiries can be directed to the corresponding author.
